# Pain situations among working adults and the educational needs identified: an exploratory survey via WeChat

**DOI:** 10.1186/s12889-019-7503-9

**Published:** 2019-08-22

**Authors:** Yajie Li, Mimi M. Y. Tse

**Affiliations:** School of Nursing, The Hong Kong Polytechnic University, Kowloon, Hong Kong, China

**Keywords:** Educational need, Pain, Working adults, WeChat

## Abstract

**Objectives:**

The aim of this study is to 1) investigate the pain situation among working adults in China; 2) explore the self-initiate pain reliving strategies applied by working adults; and 3) collect people’s interests and suggestions to the topics of the online pain education program.

**Methods:**

This is an exploratory survey through WeChat. The study was conducted from May 2018 to December 2018. Participants were recruited following the snowball sampling. In total of 664 people were recruited and 502 satisfied the criteria. SPSS was used for data analysis. Descriptive statistical analysis were used to present the utilization of pain treatments and suggested topics. Chi-square test, independent multiple logistic regression and Spearman’s correlation were used to analysis the data.

**Results:**

The overall incidence of pain among the participants is 45% and higher among female (63%) than male (37%). Neck (68.72%, 4.10 ± 2.31), shoulder (62.56%, 3.78 ± 2.41) and head (49.34%, 4.23 ± 2.52) are reported as the most common and severe pain sites. Working is affected by pain and the results show that there is a negative correlation between pain intensity and work (rs = − 0.194) among the working population. Non-pharmacological treatments (55.77%) were chosen more by pain suffers. Totally 63.39% of participants show interests in the online pain education program and physical and psychological impact of pain is the most suggested topic (22.51%).

**Conclusion:**

The pain prevalence is high among working adults in China. Impact of pain on work is a significant problem for the working adults. It is important to identify people at risk and deliver timely intervention to reduce pain. People showed their willingness in joining the online program. Therefore, future online pain education program can be developed.

## Introduction

Pain is a common and major public health problem and is negatively impact individual’s quality of life from different aspects including physical, psychological and social [1–4]. It was demonstrated that pain can restrict daily activities, decrease appetite, impair sleep and lead to depression, anxiety. Relationship strain, family burden, social isolation and even financial difficulties can be result of pain. The estimation of pain prevalence was ranging from 2 to 45% among the general population [5]. In Asia, pain among adults was reported ranging from 7.1 to 61% [6]. Study reported the pain prevalence was 35.9% in mainland China in 2016 [7]. Studies accessible in China show that the data was scarce [8].

Pain management programs had been conducted to control pain and its potential negative impact on quality of living and quality of life and effectiveness have been proved [1, 2, 9–13]. Nicholes et al. [14] used a face-to-face pain self-management program named “Manage your pain” among elderly in Australia. A copy of self-management text was provided to the participants and they were encouraged to perform the exercise and skills during the treatment session. Pain intensity, depression, pain-related disability and self-efficacy were found significantly improved.

Internet, as an innovative approach, can meet a large patient’s population with no region restriction. Educational and therapeutic programs delivered through internet is becoming increasingly popular [15]. Internet-delivered programs using the same principles, provide same evidence-based treatments and teach same skills as the face-to-face programs [1, 9, 16] but can overcome the barriers of face-to-face approach such as long distance, long waiting time, direct and indirect cost and insufficient of trained health professionals [1, 9, 17]. Dear et al. [9] evaluate the efficacy and acceptability of a validated internet-delivered chronic pain management program “The Pain Course”. The program provide therapeutic information and teaches self-management skills through internet. Results show that both physical and psychological outcomes were significantly improved with high acceptability. However, only one online pain management program is available in China which is for female teenagers to self-manage dysmenorrhea [18] even if China has more than 829 million internet users [19].

To access the internet, WeChat is a popular, free and secure mobile application which has attracted more than 900 million active users in China [20, 21]. The application includes mobile text and voice messaging communication service. Besides, it is also a platform to access internet. Spreading information and providing service can be easily achieved using WeChat.

The present study was carried out to 1) investigate the pain situation among working adults in China; 2) explore the self-initiate pain relieving strategies applied by working adults; and 3) explore people’s interests and potential topics for the future online pain management program.

## Methods

### Study design

It is a survey using self-administered questionnaire delivered through internet. Data was collected in China from May 2018 to December 2018.

### Sample and procedure

To represent the 7.8 billion working population in China with 95% confidence level and 5% margin of error, the sample size estimation of this exploratory survey was 385.

People satisfy the following criteria were regarded as eligible: 1) aged between 16 and 60; 2) have a full-time job; 3) resident of China and can understand Chinese; 4) own a smartphone which can assess the internet. Patients in hospital, experiencing a drug addiction problem or having mental illness were excluded. Participants were recruited mainly from three cities: Guangzhou, Xi’an and Xining using snowball sampling.

**Fig. 1:**
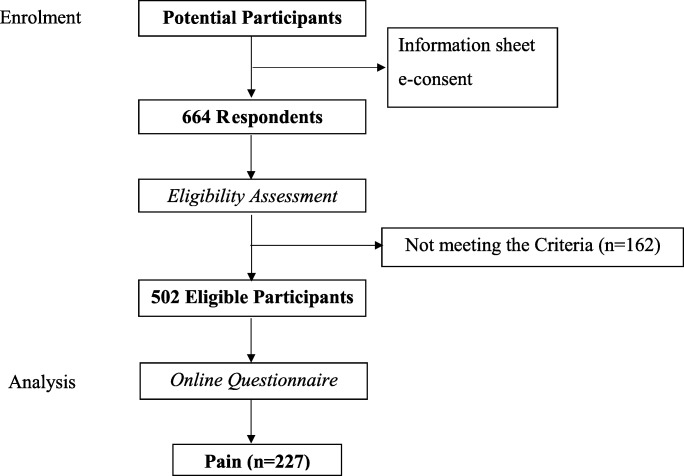
Study flow

After the ethical approval was obtained from The Hong Kong Polytechnic University, the questionnaire attached with the information sheet was first sent to a group of friends and relatives with full-time job through WeChat, and then they were asked to forward the questionnaire into different WeChat groups to recruit participants. People who completed the questionnaire were also encouraged to forward to others. All participants’ consent was collected online. After reading the information sheet, people who agree to join in were required to click “Agree” button to start the survey, otherwise they would be regarded as do not agree to participate and the online survey would be unavailable. Besides, several questions in the questionnaire were set with condition mainly in the pain situation and demographic data part, the questionnaire would be automatically and directly submitted once participants chose the choices indicate the ineligibility and this means people who are not eligible cannot finish the complete survey. The questionnaire was required to be completed online and the data was recorded at the backstage of WeChat which is accessible for the researchers which is secure. The data file can be directly downloaded into SPSS format from WeChat and the accuracy is guaranteed. Participation was voluntary as there was no remuneration for the participants. Study flow shows in Fig. 1.

### Questionnaire

The questionnaire was adopted from a previous pain management study [22] which including 5 parts:1) pain situation, 2) pain treatment, 3) public pain service and education, 4) internet accessibility and 5) demographic data. Five experts including one registered nurse, two physiotherapists, one occupational therapist and an expert of traditional Chinese medicine were invited to assess the validity using Content Validity Index for Items in which higher score indicate higher relevant. The overall Validity Index for Items was reported 0.8–1.0. Lawshe [23] suggests an index of .99 (at least), when the number of experts is 7 or less. Test-retest reliability was assessed by 5 pain suffers with two-week interval. The Intra-class Correlation Coefficient ranged from 0.802 to 0.971.

In part 1, pain was assessed by modifying and using part of Brief Pain Inventory-Short Form, a brief, self-administered questionnaire developed to assess the pain intensity, prevalence and impact [24–26]^.^ Pharmacological and non-pharmacological treatments that participants used in relieving pain in the previous period was asked in the pain treatment part. In part 2, participants were asked to score the self-perceived effectiveness of the different methods, with 0 indicating the lowest self-perceived effectiveness and 5 indicating the highest. In part 3, assessment of how participants think about the public service and education existing now was included. The source to get public service was assessed by using a multiple-choice question “where can you get the information about pain management and education?” the answers including website, leaflet, medical workers, poster, magazine and friends. The quantitative of existing public pain service and education was asked using single-choice questions: “Do you think there is enough education program / public service for pain?” Their interest and willingness towards online pain management program and the potential topics raised by participants for the future online pain education program was also asked in this part. In part 4 and 5, participants’ habit of using internet and the demographic data were collected.

### Statistical analysis

All statistical analysis was performed using Statistical Package for the Social Sciences (SPSS). Descriptive statistical analysis (n, %, mean, and standard deviation) were used to present negative impact of pain, the utilization of pain treatments and the suggested topics. Continuous data was carried out using Chi-Square test to describe the pain intensity, effectiveness of non-pharmacological treatments. To examine the association between sociodemographic factors and pain, independent multiple logistic regression was used. Spearman’s correlation was used to assess the correlation between the average pain intensity and sociodemographic factors, pain interfered activities and self-relieve strategies. A *p*-value of < 0.05 was considered statistically significant.

## Results

### Demographic characteristics

A total of 664 people answered the online questionnaire and 502 fit the criteria. Among all, 45% reported pain in the past 6 months. More female participants (63%) were suffering from pain compared with male (37%). Those aged 20 to 40 years constituted the largest group in the present study and more than 80% of the participants had a bachelor’s degree or higher education level. Details shown in Table 1.

**Table 1:** Demographic characteristics

	Total(*n* = 502) N (%)	Pain (*n* = 227, 45%) N (%)	No Pain (*n* = 275, 55%) N (%)	*p*-value	r_s_
Gender				0.272	/
F	303 (60.36)	143 (63.00)	160 (63.49)		
M	199 (39.64)	84 (37.00)	115 (45.63)		
Age				0.031^	0.077
16–20	24 (4.78)	6 (2.64)	18 (7.14)		
21–30	213 (42.43)	100 (44.05)	113 (44.84)		
31–40	179 (35.66)	75 (33.04)	104 (41.27)		
41–50	63 (12.55)	30 (13.22)	33 (13.10)		
51–60	23 (4.58)	16 (7.05)	7 (2.78)		
Marital Status				0.168	/
Married	356 (70.92)	154 (67.84)	202 (80.16)		
Single/Divorced/widowed	146 (29.08)	73 (32.16)	73 (28.97)		
Educational Level				0.034	
None	11 (2.19)	9 (3.96)	2 (0.79)		
Primary School	40 (7.97)	13 (5.73)	27 (10.71)		−0.185
Middle school	46 (9.16)	20 (8.81)	26 (10.32)		
College or Above	405 (80.68)	185 (81.50)	220 (87.30)		
Occupation				0.385	
Civil	35 (6.97)	15 (6.61)	20 (7.94)		
Profession	114 (22.71)	62 (27.31)	52 (20.63)		/
Worker	77 (15.34)	28 (12.33)	49 (19.44)		
Business/Service	90 (17.93)	38 (16.74)	52 (20.63)		
Farmer	31 (6.18)	12 (5.29)	19 (7.54)		
Producer/Transport	18 (3.59)	8 (3.52)	10 (3.97)		
Soldier	14 (2.79)	7 (3.08)	7 (2.78)		
Others	123 (24.50)	57 (25.11)	66 (26.19)		
Income (Monthly, CNY)				0.566	
< 2000	14 (2.79)	4 (1.76)	10 (3.64)		
2001–3000	22 (4.38)	10 (4.41)	12 (4.36)		−0.303
3001–4000	28 (5.58)	17 (7.49)	11 (4.00)		
4001–5000	47 (9.36)	21 (9.25)	26 (9.46)		
5001–6000	51 (10.16)	26 (11.45)	25 (9.09)		/
6001–7000	70 (13.94)	36 (15.86)	34 (12.36)		
7001–8000	88 (17.53)	39 (17.18)	49 (17.82)		
8001–9000	91 (18.13)	37 (16.30)	54 (19.64)		
9001–10,000	60 (11.95)	25 (11.01)	35 (12.73)		
> 10,000	31 (6.18)	12 (5.29)	19 (6.91)		
Living Condition				0.074	
With Family	119 (23.71)	50 (22.03)	69 (25.09)		
With Mate	115 (22.91)	52 (22.91)	63 (22.91)		
With Parents	104 (20.72)	37 (16.30)	67 (24.36)		
With Children	66 (13.15)	33 (14.54)	33 (12.00)		
Along	63 (12.55)	34 (14.98)	29 (10.55)		
With Others	35 (6.97)	21 (9.25)	14 (5.09)		

^Chi-Square test was applied, *p*-value < 0.05 is considered statistically significant

### Pain sites, intensity and interference

**Fig. 2:**
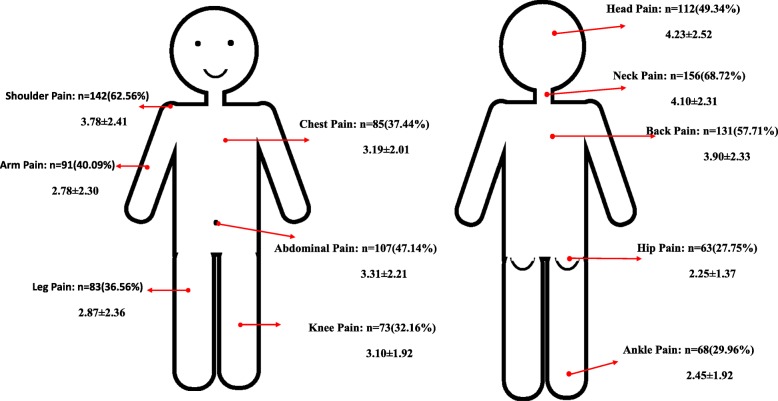
Pain sites and pain intensity

Neck is reported as the most common pain site (68.72%) followed by shoulder (62.56%) and back (57.71%) (Fig. 2). Head (49.34%) is the fourth common pain sites reported with highest pain score (Mean = 4.23, SD = 2.52). The pain score at neck, back and shoulder are 4.10 ± 2.31, 3.90 ± 2.33 and 3.78 ± 2.41 respectively.

Table 2 shows the pain interference. Mood (56.4%), working (46.7%) and daily activities (37%) are most commonly been impacted by pain. Significant correlation is shown between pain intensity and mood (*r* = − 0.148, *p* < 0.05), working (*r* = − 0.194, *p* < 0.01), sports (*r* = − 0.156, *p* < 0.05) and entertainment (*r* = − 0.131, *p* < 0.05).

**Table 2:** Negative impact of pain

Items	N (%)	*p*-value	r^a^
Mood	128 (56.4)	0.026	−0.148^b^
Working	106 (46.7)	0.003	−0.194^c^
Daily activities	84 (37.0)	0.367	−0.060
Sleep	73 (32.2)	0.232	−0.079
Sports	73 (32.2)	0.018	−0.156^b^
Relationship	41 (18.1)	0.863	−0.011
Entertainment	37 (16.3)	0.048	−0.131^b^

^a^Pearson correlation is used

^b^Correlation is significant at the 0.05 level (2-tailed)

^c^Correlation is significant at the 0.01 level (2-tailed)

### Pain relieve methods and self-perceived effectiveness

Results show that 20.26% participants did not take any actions, whereas their pain score is the highest 3.28 ± 2.64 on an 11-point scale (Table 3). Non-pharmacological treatments were more chosen (56.39%) than pharmacological treatments (23.35%). Lying down (60.29%), massage (58.82%) and hot compress (44.85%) are the top three used non-pharmacological strategies. Lying down was reported with the highest SPF score (Mean = 3.76, SD = 2.68) followed by hot compress (Mean = 3.15, SD = 2.79) and massage (Mean = 2.93, SD = 2.64).

**Table 3:** Pain relieve methods used by pain suffers and the self-perceived effectiveness of the treatments

	N (%)	Pain score (Mean ± SD)	Self-perceived effectiveness (Mean ± SD)	r
Pain relieve methods				−0.339
Do not take any treatments	46 (20.26)	3.28 ± 2.64	–	
Non-pharmacological treatments	128 (56.39)	2.59 ± 0.72	–	
Lying down	82 (60.29)	–	3.76 ± 2.68	
Hot Compress	61 (44.85)	–	3.15 ± 2.79	
Massage	80 (58.82)	–	2.93 ± 2.64	
Deep Breath	49 (36.03)	–	2.92 ± 2.83	
Sports	55 (40.44)	–	2.85 ± 2.52	
Cupping	55 (40.44)	–	2.78 ± 2.65	
Plaster	46 (33.82)	–	2.70 ± 2.61	
Scrapping	46 (33.82)	–	2.70 ± 2.53	
Acupuncture	42 (30.88)	–	2.55 ± 2.50	
Reading	42 (30.88)	–	2.53 ± 2.63	
Cold Compress	43 (31.62)	–	2.52 ± 2.55	
Talking	40 (29.41)	–	2.52 ± 2.66	
Music	44 (32.35)	–	2.51 ± 2.47	
Aromatherapy	37 (27.21)	–	2.38 ± 2.46	
Pharmacological treatment	53 (23.35)	2.19 ± 1.87	–	

*N* = 227, Total No. of Non-Pharmacological treatments use = 136

*SD* Standard deviation

r is calculated using Pearson correlation

Guideline:

Small *r* = 0.10 to 0.29

Medium *r* = 0.30 to 0.49

Large *r* = 0.50 to 1.0

### Willingness to the online pain education program and the potential topics of the program raised by participants

Around 64% of respondents showed interest and willingness in joining the online pain education program. Seventy-four percent respondents would like to learn the influence of pain. Together with pharmacological treatment (71.74%) and relationship between pain and disease (67.39%) were found to be the most attractive topics (Table 4).

**Table 4:** Potential topics of online education program raised by participants

Topics	N	%
Physical and psychological impact of pain	237	73.60
Pharmacological treatment	231	71.74
Relationship between pain and diseases	217	67.39
Non-pharmacological treatment	188	58.39
Definition of pain	180	55.90

*N* = 322, Total No. of responses to the topics = 1053

## Discussion

Totally 502 participants were studied with 60% of female participants. Most of the participants were aged between 21 and 40. Pain prevalence was reported 45% among the working adults. 64% participants showed willingness in joining in the online pain education program and participants’ educational needs were identified.

The present study reported a high prevalence of pain (45%) among working adults. The pain prevalence is higher than that of the general population in China which was reported 35.9% in the previous study [7]. Neck, shoulder and back are the most commonly reported pain sites in the present study with high pain score. The results may result by the office automatization policy in China. With the development of the technology and the environment protection nowadays, companies which advocate office automatization are increasing [27]. People are expected to work on the computer, in regard of this policy, long sitting time and computer-facing time are becoming significant factors causing pain especially in neck, shoulder and back among the working adults.

Significant correlation between pain intensity and educational level and income were demonstrated in the present study. The findings are consistent with previous studies. Researches had indicated that the higher risk of pain is correlated with lower sociodemographic status [7, 9, 28]. As it reported by National Bureau of Statistics of China, the average monthly income of Chinese was 2165 Yuan (318 USD) in 2017 and lower in rural area [29]. Accessible medical resources are limited in the rural area and the average education level is lower as well. These restrictions can indirectly lead to a high pain incidence and intensity.

Work is impacted by pain according to the results which is a significant problem especially for the working adults. In China, salary is related with the attendance. Longer time asking for leave would lead to lower salary. Due to the lower socioeconomic status, working adults would experiencing more severe pain and the overall quality of life would be impacted. Managing pain is momentous among working adults.

Around one fourth of the participants did not take any actions to manage their pain situation, and this may lead to persistent and worse pain. Non-pharmacological treatments are more chosen perhaps is on account of lacking of time to see doctor, insufficient medication knowledge and the convenient and easy accessibility with low cost. Lying down, hot compress and massage were among the most common non-pharmacological treatments applied and the self-perceived effectiveness were high. Researchers had indicated that use of hot water bag is common and it is an effective methods in pain management [18, 30, 31]. Lying down and massage can be easily self-initiated to relieve pain. Since this kind of pain-relieving strategies are easy and convenient to use, they can be recommended to the working adults.

The patients’ demand for pain management programs in China was high. Chu et al. [32] reported a 10-year outcome of pain management program for Chinese suggested that the recurrence of body pain was mainly due to lack of effective pain management programs. In this way, pain management programs are needed. Considering the applicability and advantages of internet together with participants educational needs identified, online pain education programs can be developed. The topics raised by the participants should be involved in the future pain education program.

There are several limitations of this study should be noted. First, the participants’ recruitment was following the snowball sampling which was reported may be more biased toward the more cooperative participants who are willing to participate in the study [33]. In regard of this, most of the participants are mainly from three cities which may be less representative to the nationwide working population. In addition, the participants gathered more in age group of 20 to 40 years may result of the internet-based recruitment as the internet is more popular among younger population. As the consequence of the younger age group, the pain prevalence reported may be lower than reality, therefore, stratified analyses of pain by sociodemographic factors should be made in the future study.

This study has a number of strengths in spite of the limitations. Firstly, it is the first time to use WeChat as an approach to deliver online survey in China. Secondly, this study specially focus on the working adults which is meaningful in learning pain situation in the specific group. The results contribute to the society to evaluate the overall pain situation. Thirdly, the educational needs of the pain suffers were identified and their willingness in joining the future online program were collected.

### Conclusion

The pain prevalence is high among working adults in China. Impact of pain on work is a significant problem for the working adults. It is important to identify people at risk and deliver timely intervention to reduce pain. People showed their willingness in joining the online program. Therefore, future online pain education program can be developed.

## Data Availability

The datasets used and/or analyzed during the current study are available from the corresponding author on reasonable request.
